# A Longitudinal Study of the Relations Between Theory of Mind, Executive Function, and Lying in Children

**DOI:** 10.3389/fpsyg.2021.766891

**Published:** 2021-12-10

**Authors:** Changzhi Zhao, Siyuan Shang, Alison M. Compton, Genyue Fu, Liyang Sai

**Affiliations:** ^1^Department of Psychology, College of Education, Hangzhou Normal University, Hangzhou, China; ^2^Center for Cognition and Brain Disorders, The Affiliated Hospital of Hangzhou Normal University, Hangzhou, China; ^3^Department of Psychology, University of California, San Diego, La Jolla, CA, United States

**Keywords:** children, lying, theory of mind, executive function, longitudinal

## Abstract

This study used longitudinal cross-lagged modeling to examine the contribution of theory of mind (ToM), executive function (EF) to children’s lying development and of children’s lying to ToM and EF development. Ninety-seven Chinese children (initial *M*_age_ = 46 months, 47 boys) were tested three times approximately 4 months apart. Results showed that the diverse desire understanding and knowledge access understanding components of ToM, as well as the inhibitory control component of EF predicted the development of children’s lying, while the diverse belief understanding and false belief understanding components of ToM, and the working memory component of EF did not predict development of children’s lying. Meanwhile, children’s lying predicted development of children’s belief-emotion understanding components of ToM, but not any other ToM components, or EF components. These findings provide longitudinal evidence for the relation between ToM, EF, and children’s lying during the preschool years.

## Introduction

Attempts to understand the development of lying have often treated lying as a moral problem (Hartshorne and May, 1928; Piaget, 1932). However, it is being increasingly recognized that it is also a cognitive problem (Lee, 2013). Specifically, links have been investigated between the cognitive skills of theory of mind (ToM), which involves the ability to understand other’s actions in terms of mental states (Wellman, 1990), and of executive function (EF), which involves the ability to guide goal-oriented behavior under conscious control (Zelazo and Carlson, 2012). As reviewed below, there have been many studies documenting these relations, but prior work on this topic has generally relied on cross-sectional assessments. Consequently, the nature of these relations is unclear. The present study seeks to fill this gap in the literature by looking at these links longitudinally.

Children start to tell lies at around 2.5 years old (Russell et al., 1991; Evans and Lee, 2013; Fu et al., 2018). During the preschool period, the tendency of children’s lies increase with age (Talwar and Lee, 2008; Evans et al., 2011). ToM and EF are two key cognitive factors, which have been suggested to play an important role in children within this age range. ToM has been suggested to be related to children’s lying. Specifically, Talwar and Lee (2008) proposed a hypothesis regarding the relation between ToM and children’s lying, which suggests that children’s lying is related to first-order false belief understanding of ToM, because to lie successfully, one must instill a false belief into other’s mind (Talwar and Lee, 2008; Talwar and Crossman, 2011). In line with this argument, prior work has shown there are positive relations between false belief understanding and children’s lying (e.g., Lewis et al., 1989; Hala et al., 1991; Fu et al., 2018; Lee and Imuta, 2021). This hypothesis has been recently extended and suggests that lying is related to early ToM understanding that develops before false belief understanding among young children who are not able to pass false belief understanding. These early ToM includes diverse desire (understanding that others could have different desires than they do), diverse belief (understanding that others could have different beliefs than they do), knowledge access (understanding that someone can have knowledge of a fact that other people do not have), and belief-emotion (understanding how a person will feel, given a belief that is mistaken; Wellman and Liu, 2004). In line with this extended hypothesis, Ma et al. (2015) and Leduc et al. (2017) found evidence for the importance of knowledge access in children’s lying in 2.5- to 3-year-old. In addition, Sai et al. (2020) found evidence that both diverse belief and belief-emotion were positively correlated with children’s lying in 3-year-old.

In addition to ToM, EF has also been proposed to play an important role in children’s lying. EF is a group of higher order cognitive processes, which includes distinct subcomponents, such as inhibitory control, working memory, and cognitive flexibility (e.g., Austin et al., 2014). Several theories of deception such as interpersonal deception theory (Buller and Burgoon, 1996) and the activation–decision–construction model (ADCM, Walczyk et al., 2003) have proposed that inhibitory control and working memory are particularly related to lying because to tell a lie, children need to suppress the prepotent tendency to tell the truth (Evans and Lee, 2013), while also holding the truth and creating its alternatives in their minds (Alloway et al., 2015). This argument has been supported by findings showing positive relations between children’s lying and their inhibitory control, and working memory ability (Carlson et al., 1998; Talwar and Lee, 2008; Williams et al., 2017, for a meta-analysis, see Sai et al., 2021).

Although many studies have provided evidence about hypotheses regarding relations between children’s lying and their ToM (*ToM hypothesis*), and between children’s lying and their EF (*EF hypothesis*), most of the existing evidence are from cross-sectional studies. Almost no longitudinal study has been conducted to test these two hypotheses. In addition, there are open questions about the nature of these relations. One possibility is that children’s ToM and EF support the development of children’s lying. In line with this possibility, Ding et al. (2015) found that training 3-year-old children who are unable to tell lies to learn about mental-states concepts makes them begin to tell lies, and children who have better ToM and EF are faster at learning how to deceive (Ding et al., 2017). However, evidence indicating that training children to deceive leads to improvements in their ToM and EF raises the possibility that children’s lying supports their cognitive abilities (Ding et al., 2018).

To date, there is only one longitudinal study examining the nature of the associations between children’s lying and their ToM, and EF (Talwar et al., 2019). Specifically, Talwar et al. (2019) found children’s tendencies to tell lies were unrelated to ToM or EF. However, it should be noted that children in their research were 4.65 years old on average when first tested. This is relevant because at around this age almost all children likely have sufficient cognitive ability to tell lies, which may be why individual differences in these capacities were not predictive. Of interest in our research was to test children multiple times between the ages of 3- to 4-years of age, when ToM skills and EF are rapidly developing. We also expand upon finding of Talwar et al. (2019) by including all ToM components on the Wellman and Liu scale instead of just false belief understanding.

In summary, the present study is the first study to examine development of lying and its relation to ToM and EF longitudinally by looking at multiple time points between 3 and 4 years of age. We hypothesized that children’s ToM and EF would predict development of children’s lying. Specifically, based on the two hypotheses, we expected that, children’s ToM such as diverse belief, knowledge access, and false belief understanding would predict children’s lying (e.g., Leduc et al., 2017; Fu et al., 2018; Sai et al., 2020), and we also expected that children’s inhibitory control and working memory would predict children’s lying (Hala and Russell, 2001; Talwar et al., 2017b).

## Materials and Methods

### Participants

We conducted a prior Power analysis using G*Power 3.1 with Power (1-β) set at 0.80 and *α* = 0.05, to determine the required sample size (Faul et al., 2009). Results showed that 98 participants were required to detect the contribution of ToM, EF to children’s lying development in several linear multiple regression with a medium effect size (effect size *f*^2^ = 0.15). We recruited 97 participants in an eastern city of Mainland China at the first test. All participants are Han Chinese preschool children. Recruited children came from families in a diverse range of socioeconomic status (e.g., working-, middle-, and upper-class), with the majority from middle-class backgrounds. They were tested at three time points approximately 4 months apart (Time1, Time2, and Time3), and the initial test began in 2018. We selected this relative short time interval because 3 and 4 years of age are known to be a period of a rapid development for children’s lying. At Time1 (T1), there were 97 children (47 boys, *M*_age_ = 46.12 months, *SD* = 3.12, range 39–51 months). At Time2 (T2), there were 93 children remaining (44 boys, *M*_age_ = 50.70 months, *SD* = 3.05, range 44–55 months). Four children were excluded at T2 because they did not finish the procedure. At Time3 (T3), there were 91 children remaining (43 boys, *M*_age_ = 54.10 months, *SD* = 3.04, range 47–58 months). Two additional children were excluded at T3 because they did not finish the procedure. A total of 89 children had completed all tasks at all three time points. The informed consent of parents or guardians and participants’ verbal assents were obtained prior to their participation in the study. This study passed through the university ethics review board.

### Procedure

At each time point, participants completed one lying task, one ToM task, and two EF tasks. The order of these measures was counterbalanced across participants. At T1, participants also completed verbal ability test after completing all other tests.

#### Lie-Telling Behavior Task

We used a hide-and-seek task to examine children’s lying (e.g., Ding et al., 2017). On this task, the child hid a prize while an experimenter covered his eyes and provided information on the location of the prize when the experimenter looked for it. The procedure included a training phase followed by a test phase, and stickers were used as prizes. On the training phase, children were first taught the rules of the game. Specifically, they played a no-deception hiding game with the experimenter in which they hid a sticker in one of two cups, and the experimenter then guessed which cup the sticker was in. The child won if the experimenter guessed incorrectly, and lost if the experimenter guessed correctly. All of the children were able to follow the rules correctly after two or three trials.

During the test phase, children had the opportunity to win stickers if they engaged in lying with the experimenter. Specifically, they were told that they would lose the sticker on each trial where the experimenter found the sticker, and kept the sticker if the experimenter did not find it. Children were asked to choose 10 favorite stickers from a set of stickers and were told that they could keep all these stickers if he or she win. On each of 10 test trials, the child hid a sticker while the experimenter closed her eyes. After the child announced that sticker was hidden, the experimenter opened her eyes and asked, “Where did you hide the sticker?” The child then indicated a specific cup, and the experimenter always guessed the cup that the child had indicated. The dependent measure was whether the child tried to mislead the experimenter by indicating the wrong location. Each time the child did this, they received one point, which allowed them to earn between 0 and 10 points.

#### Theory of Mind Task

We used a Chinese version of the ToM scale to examine children’s ToM understanding. The Chinese version is the same as the North American version except that the names of the characters and objects are changed to ones that are familiar to Chinese children. This scale includes five subtasks: diverse desire, diverse belief, knowledge access, false belief, and belief-emotion (Wellman and Liu, 2004; Ding et al., 2015). For each task, the experimenter told the child a story and then the child answered two questions: a warm-up question along with a target question. If a child answered both questions correctly, he or she received one point, which indicating that the child could understand the certain component of ToM. We used three equivalent versions of this scale. The first two sets were the same as that used by Ding et al. (2015). The third version, which was adapted, had adequate internal consistency with the first two set, with Cronbach’s alpha ranging from 0.60 to 1.00.

#### Executive Function Tasks

Children’s inhibitory control was assessed using a revised computer-based Flanker-Fish task (Diamond et al., 2007). In the task, children were asked to feed the target fish according to different rules, and fish were presented on the screen until children responded. This task consists of two phases: Blue Fish and Pink Fish. Children were asked to only pay attention to the hungry fish (the target) while ignoring the other fish. Children were instructed to press the left or right arrow keys in the direction where the hungry fish was facing (left or right) in order to feed it. On the Blue Fish phase, the hungry fish was always the middle one, and on the Pink Fish phase, it was on the left and right sides.

We recorded participants’ reaction time (RT) in each correct trial and their reaction accuracy (ACC) in each session. To take into account both reaction time and reaction accuracy, we used the inverse efficiency scores (IES; = RT/ACC) of Pink Fish trials to score inhibitory control (Ding et al., 2014). Note that higher IES scores reflect lower inhibitory control.

Children’s working memory was assessed using the Digit-Span task (e.g., Evans and Lee, 2011). Participants were told a sequence of digits which they had to verbally repeat in the same or reverse order (the Digit-Span forward or backward task). They were allowed two attempts on each of the eight trials (dependently of the success in the first attempt), and the test was terminated when the child failed both attempts on any of the trials. The dependent variable was the total number of trials that had been successful repeated on Digit Span Forward and Digit Span Backward. Each correctly repeated trial was awarded one point.

#### Verbal Ability Task

To measure children’s receptive verbal ability, we used Chinese version of Peabody Picture Vocabulary Test-Revised (PPVT-R; Sang and Liao, 1990; Carlson and Moses, 2001). The child listened to a word uttered by the interviewer and then selected one of four pictures that best describes the word’s meaning. Testing begins with a starting point that is based on the child’s age and proceeds until the child has incorrectly identified six of eight consecutive items. The raw score can be calculated by subtracting the number of errors from the total ceiling score. The Chinese version of PPVT is the same as the North American version except that certain words and pictures that are not familiar to Chinese children were changed to familiar ones.

#### Data Analysis Plan

Following descriptive analyses for each variable, repeated-measures ANOVAs were conducted to examine time (T1 vs. T2 vs. T3) effects for the variables. To examine the relation between children’s lying and their ToM and EF, we performed partial correlations among these variables, controlling for children’s age and verbal ability.

To further explore the longitudinal associations between children’s lying and ToM and EF across time, cross-lagged path models were conducted using MPlus 8 (Muthén and Muthén, 1998-2012), with four competing path models (Casper et al., 2019, see Supplementary Figure S1; Supplementary Material): a stability model (M1), a ToM/EF-to-lying model (M2), a lying-to-ToM/EF model (M3), and a reciprocal model (M4). The stability model included only temporal stabilities (i.e., paths between corresponding variables for each possible pair of data collection waves) and synchronous correlations (i.e., correlations between different variables measured at the same time). The M2 was based on the stability model, but included cross-lagged paths between ToM, EF, and subsequent lying behavior. The M3 was also built on the stability model and additionally included cross-lagged paths between lying behavior and subsequent ToM/EF. Finally, the reciprocal model included cross-lagged paths between ToM, EF, and subsequent lying behavior as well as cross-lagged paths between lying behavior and ToM, EF. In all four models, we used a maximum-likelihood estimation (ML), and paths from children’s age and verbal ability (measured at T1) to all predicted variables were added to control the influence of age and verbal ability. The competing nested models were compared *via* chi-square difference tests. Because all models included three waves of data collection and thus two separate time lags, we were able to test our hypotheses with respect to the time lag between T1 and T2, and between T2 and T3.

## Results

Descriptive statistics for each test (mean and standard deviations) at each time point were presented in Table 1. Repeated-measures ANOVAs revealed significant main effects of time across these variables. With time, children’s lying tendency increased, and their understanding of each component of ToM and performance on each component of EF improved (see Table 1).

**Table 1:** Descriptive statistics of assessed variables for the three measurement time points.

Variable	T1	T2	T3	F	*Ranges*
	*M (SD)*	*M (SD)*	*M (SD)*		
Lying	4.55 (4.56)	6.21 (4.60)	8.43 (3.13)	32.31^***^	0–10
**Theory of mind**
Diverse desire	0.91 (0.29)	0.97 (0.18)	0.99 (0.11)	3.65^*^	0–1
Diverse belief	0.79 (0.41)	0.94 (0.23)	0.92 (0.27)	6.28^**^	0–1
Knowledge access	0.33 (0.47)	0.60 (0.49)	0.89 (0.32)	53.12^***^	0–1
False belief	0.10 (0.30)	0.30 (0.46)	0.51 (0.50)	19.99^***^	0–1
Belief-emotion	0.34 (0.48)	0.51 (0.50)	0.65 (0.48)	11.56^***^	0–1
**Executive function**
Working memory	5.42 (1.29)	5.78 (1.43)	6.49 (1.98)	20.22^***^	0–16
Inhibitory control	5408.93 (3657.81)	4180.69 (2303.50)	3222.23 (1608.44)	20.77^***^	
Verbal ability	37.09(16.47)				0–175

Inhibitory control = the inverse efficiency score (IES) of Pink Fish in the Flank-Fish task.

*** *p* < 0.001;

** *p* < 0.01;

* *p* < 0.05.

### Partial Correlations Between Children’s Lying and Cognitive Factors

Partial correlation analyses showed that, after controlling for children’s age and verbal ability at T1, children’s lying at T1 was significantly associated with their belief-emotion at T1, *r(93)* = 0.27, *p* = 0.009, and inhibitory control at T1, *r(93)* = −0.21, *p* = 0.043; children’s lying at T2 was significantly associated with their diverse desire at T1, *r(89)* = 0.25, *p* = 0.017, diverse belief at T2, *r(89)* = −0.25, *p* = 0.016, belief-emotion at T3, *r(85)* = −0.28, *p* = 0.009; children’s lying at T3 was significantly associated with their diverse desire at T1, *r(87)* = 0.26, *p* = 0.014, knowledge access at T2, *r(87)* = 0.30, *p* = 0.004, and inhibitory control at T2, *r(87)* = −0.24, *p* = 0.022, and inhibitory control at T3, *r(85)* = −0.25, *p* = 0.018. Given these significant relations between children’s lying and their cognitive abilities in different time points (see Table 2), we further conducted cross-lagged path models to explore the direction of these associations.

**Table 2:** Partial correlations between children’s lying and cognitive abilities.

Variables	T1 (Lying)		T2 (Lying)		T3 (Lying)	
	T2	T3	T1	T3	T1	T2
**Theory of mind**						
Different desire	−0.13	0.08	0.25^*^	0.13	0.26^*^	0.01
Different belief	−0.10	0.07	0.09	−0.07	0.14	−0.12
Knowledge access	0.06	0.19	0.18	0.05	0.09	0.30^**^
False belief	0.10	0.02	0.12	0.05	0.15	0.13
Belief-emotion	0.13	−0.07	−0.03	−0.28^**^	0.12	−0.06
**Executive function**						
Working memory	0.12	−0.09	0.07	−0.12	−0.05	0.19
Inhibitory control	−0.14	−0.04	0.02	−0.19	−0.13	−0.24^*^

Partial correlation after controlling for verbal ability and children’s age at T1.

** *p* < 0.01;

* *p* < 0.05.

### Reciprocal Relations Between Lying and ToM/EF

Cross-lagged path analyses showed that all the four models had an acceptable fit to the data, and Model 4 which included cross-lagged paths in both directions had an excellent fit, χ^2^(52) = 70.258, *p* = 0.047, χ^2^/df = 1.351, CFI = 0.890, RMSEA = 0.063. And the Model 4 fit the data significantly better than the stability model (M1), *Δ*χ^2^(5) = 22.984, *p* < 0.01, and the ToM/EF-to-lying model (M2), *Δ*χ^2^(1) = 6.273, *p* < 0.05, and the lying-to-ToM/EF model (M3), *Δ*χ^2^(4) = 16.719, *p* < 0.01. The model-fitting results are shown in Table 3. Results showed that diverse desire positively predicted development of children’s lying from T1 to T2, while knowledge access and inhibitory control might have a marginally positive impact on the change of children’s lying from T2 to T3. Surprisingly, children’s lying negatively predicted development of belief-emotion from T2 to T3 (see Supplementary Figure S2 for detailed descriptions).

**Table 3:** Fit indices for competing models and results of chi-square difference tests.

	χ^2^	*Df*	χ2/*df*	CFI	RMSEA	Δχ^2^	*Δdf*
Stability model (M1)	93.242	57	1.636	0.782	0.085		
ToM/EF-to-lying model (M2)	76.531	53	1.444	0.858	0.071	16.711^**^^a^	4
Lying-to-ToM/EF model (M3)	86.977	56	1.553	0.813	0.079	6.265^*^^a^	1
Reciprocal model (M4)	70.258	52	1.351	0.890	0.063	22.984^**^^a^	5
						6.273^*^^b^	1
						16.719^**^^c^	4
Reciprocal model (M5)	42.072	31	1.357	0.923	0.063	28.186^d^	21

CFI, comparative fit index; RMSEA, root mean square error of approximation.

a In comparison with Model 1.

b In comparison with Model 2.

c In comparison with Model 3.

d In comparison with Model 4.

** *p* < 0.01;

* *p* < 0.05.

**Figure 1:**
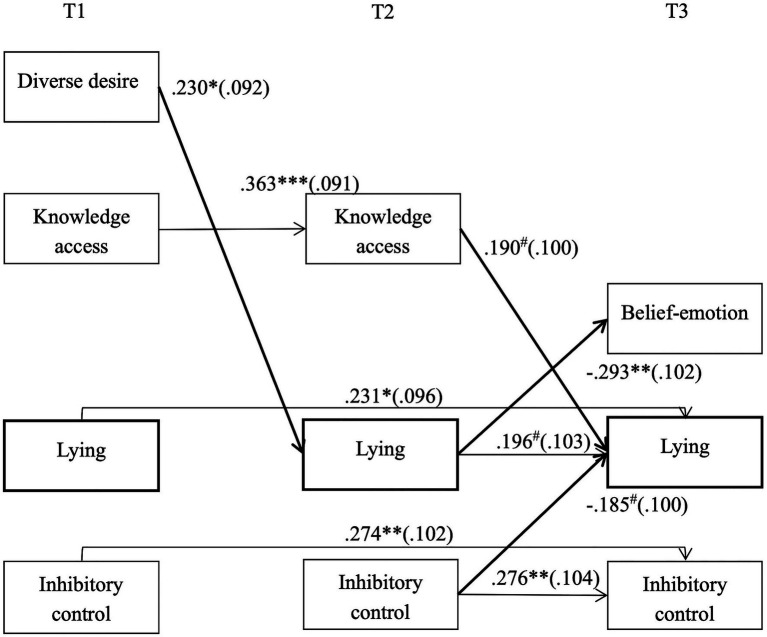
Standardized estimates for significant paths in the reciprocal model after controlling for children’s age and verbal ability. Synchronous correlations are omitted from this figure, but were included in the analyses. ^***^p < 0.001; ^**^p < 0.01; ^*^p < 0.05; and ^#^*p* < 0.08.

Considering limited sample size, we computed the final model, Model 5, which omitted nonsignificant paths from Model 4, to test our hypotheses, χ^2^(31) = 42.027, *p* = 0.089, χ^2^/*df* = 1.357, CFI = 0.923, RMSEA = 0.063, and the results remained the similar (also see Figure 1). Results showed that diverse desire positively predicted development of children’s lying from T1 to T2 (*β* = 0.230, SE = 0.092, *p* = 0.012), while knowledge access (*β* = 0.190, SE = 0.100, *p* = 0.056) and inhibitory control (*β* = −0.185, SE = 0.100, *p* = 0.063) have a marginally positive impact on the development of children’s lying from T2 to T3. Meantime, children’s lying negatively predicted development of belief-emotion from T2 to T3 (*β* = −0.293, SE = 0.102, *p* = 0.004).

## Discussion

Prior research has documented relations between children’s lying and their ToM, and between children’s lying and their EF. The present research builds on that work to clarify the nature of these associations by looking at these relations longitudinally over a period of about 8 months. We found that children’s understanding of diverse desires significantly predicted their developing lies. Both knowledge access and inhibitory control also predicted their developing lies, but only with a marginal significant. Children’s lying negatively predicted their developing belief-emotion understanding.

In line with ToM hypothesis, we found that diverse desire understanding predicted development of children’s lying. The understanding of diverse desire refers to understanding that others have different desires than they do (Wellman and Liu, 2004). In this study, children were more likely to mislead the experimenter when they understood that the experimenter wanted the stickers by himself. These results extend previous cross-sectional findings and provide longitudinal evidence of ToM supports children’s lying. We also found that knowledge access understanding marginally predicted development of children’s lying. The understanding of knowledge access refers to understanding that others do not have knowledge of a fact that ones have (Wellman and Liu, 2004; Leduc et al., 2017). In the hide and seek task, children were more likely to mislead the experimenter by pointing the empty cup when they understood that the experimenter did not know true location of stickers as theirs because they closed their eyes at that time. This result is consistent with findings from Ma et al. (2015) and Leduc et al. (2017), which they found that children’s understanding of knowledge access is related to children’s lying to conceal their rule-violation in a temptation resistance paradigm. It should be noted that Leduc et al. (2017) also examined diverse desire understanding and children’s lying, but fail to find a significant relation. One possible reason is that lying task in the current study requires more diverse desire understanding than temptation resistance paradigm used by Leduc et al. (2017). Interestingly, these two ToM components predicted children’s lying at different time points. Specifically, over time there is a shift from early-developing ToM components such as diverse desires predicting children’s lying to relatively late-developing ToM components such as knowledge access predicting children’s lying. One possible explanation is that lies young children tell are basic and inconsequential (e.g., wish fulfillment), which early ToM can support. However, with age, children’s lying becomes relatively complex, which may need high-level ToM to support (Talwar and Lee, 2008). Further studies should add measures that could assess lying strategies that children use to examine this hypothesis.

It should be noted that a number of previous studies found that children’s lying was related to their false belief understanding (e.g., Ding et al., 2015; Fu et al., 2018). However, the present study did not find a relation between false belief understanding and children’s lying. One possible reason is that children in our study were relatively young (3.8 years in Time 1 and 4.1 years in Time 2), and scored poorly on tests of false belief understanding (10% of children in Time 1, and 30% of children in Time 2 passed the first belief test). Our results are consistent with studies, which used a similar age range as our study also did not find a relation between false belief understanding and children’s lying (Xu et al., 2005; Talwar et al., 2017a). However, studies who found the relation between false belief understanding and children’s lying often included children above 5 years old (Talwar and Lee, 2008). Further studies should include older children to examine this hypothesis.

Consistent with prediction based on ADCM (Walczyk et al., 2003), we found that inhibitory control supported development of children’s lying. According to ADCM, the activation of the truth occurred automatically, to lie; children need suppress the prepotent tendency to tell the truth (Evans and Lee, 2013). It should be noted that the relation between children’s lying and inhibitory control is marginally significant. Some of previous studies have found a significant relation between inhibitory control and children’s lying (Talwar and Lee, 2008; Evans and Lee, 2013; Fu et al., 2018), but the tasks used in those studies not only measure inhibitory control ability but also measure working memory (Talwar and Lee, 2008; Evans and Lee, 2013), or cognitive flexibility (Fu et al., 2018). In the same vein, to successfully lie about the whereabouts of the hidden sticker in the task, children not only need to inhibit the prepotent tendency to report the true location of the sticker but also need to switch to a response that is inconsistent with the true state of affair (i.e., the empty hand). Thus, it is possible that inhibitory control and cognitive flexibility together support children’s lying in our paradigm (Ding et al., 2018; Fu et al., 2018). Thus, the task that only measured inhibitory control ability in this study may lead this marginal effect. Further studies should consider using tasks measuring both inhibitory control and cognitive flexibility to examine this question. Inconsistent with the EF hypothesis, we did not find a relation between children’s lying and their working memory. One possible reason may be related to our task. Specifically, children can see where the sticker hid when telling a lie to mislead the experimenter, which does not require much working memory load. It is also possible that working memory may play more important role in strategic lying. For example, a few studies have shown that working memory is related to children’s ability to maintain their lies, but not relate to their initial lies (Evans and Lee, 2011; Alloway et al., 2015).

Unexpectedly, we found that children lying in Time 2 negatively predicted development of children’s belief-emotion in Time 3. Further research will be needed to see whether this finding replicates. If it does, one possible explanation is that children who lied less are more likely to have concerns about the experimenter feeling badly about losing which may help them to advance their understanding of belief-emotion.

There are several limitations in the present study. First, the present study only examined the relations between children’s lying and cognitive factors such as ToM and EF. Further longitudinal work should be done that also examines the role of social factors such as family factors (e.g., parenting styles) and peer relationship. Second, the present study only focused on children with 3–4 years old, further studies should also include a broader age range to examine the relation between children’s lying and ToM, and between children’s lying and EF. Third, the sample size of the present study is relatively small, further studies with larger sample size are needed to examine these issues. Lastly, further studies should also examine lying among children with ToM or EF impairments (Maoz et al., 2014; Nuria et al., 2016) to test the contribution of ToM/EF on children’s lying.

Although previous research has indicated that ToM and EF play an important role in development of children’s lying, but most of the studies used cross-sectional design and thus are not able to test the hypothesis that whether ToM and EF support development of children’s lying. This study uses longitudinal design and provides evidence for the above question. It should be noted that the development of children’s lying could also be influenced by culture. For example, several studies have shown that children in China have better EF skills than children in United States (Sabbagh et al., 2006), thus it is possible that children in China may learn to lie earlier than children in United States. Second, previous research also shows that there are some cultural differences in tendency of children’s lying. For example, Chinese children were more inclined to choose lying for group’s benefit than Canadian children because Chinese children were more group-oriented while Canadian children were more individual-oriented (Fu et al., 2007). Thus, it is possible that Chinese children are more likely to tell lies for group’s benefit than children in Canadian.

In summary, the present study used a longitudinal method to examine the nature of the relations between children’s lying and their ToM and EF. We found that ToM components of diverse desire predicted development of children’s lying. Both knowledge access and inhibitory control predicted children’s lying with a marginal significant level. These findings provide the first longitudinal evidence that which components of ToM are most associated with lying change over time.

## Data Availability Statement

The raw data supporting the conclusions of this article will be made available by the authors, without undue reservation.

## Ethics Statement

The studies involving human participants were reviewed and approved by Department of Psychology, College of Education, Hangzhou Normal University. Written informed consent to participate in this study was provided by the participants’ legal guardian/next of kin.

## Author Contributions

CZ searched literature, collected the data, performed the data analysis, and wrote the original draft. SS made contributions to data analysis and funding acquisition, and revised the article. AC revised the article. GF made contributions to funding acquisition and revised the article. LS generated the research concept, contributed to funding acquisition, and revised the article. All authors contributed to the article and approved the submitted version.

## Funding

This study was supported in part by grants from the National Natural Science Foundation of China (U1736125 and 32071068) and from the Cultivation Project of Provincial Characteristic Key Discipline in the College of Education of Hangzhou Normal University (20JYXK003 and 20JYXK038).

## Conflict of Interest

The authors declare that the research was conducted in the absence of any commercial or financial relationships that could be construed as a potential conflict of interest.

## Publisher’s Note

All claims expressed in this article are solely those of the authors and do not necessarily represent those of their affiliated organizations, or those of the publisher, the editors and the reviewers. Any product that may be evaluated in this article, or claim that may be made by its manufacturer, is not guaranteed or endorsed by the publisher.
