# Supplementary material for: A Longitudinal Study of the Relations Between Theory of Mind, Executive Function, and Lying in Children
Source: Front Psychol. 2021 Dec 10;12:766891. doi: 10.3389/fpsyg.2021.766891 (PMC8703068; doi:10.3389/fpsyg.2021.766891)
Supplement: Supplementary file 1 [file Table_1.DOCX]

# *Supplementary Material*

# Supplementary Data

Results of Model 4 showed that diverse desire positively predicted development of children’s lying from T1 to T2 (β = .230, SE = .092, *p* = .012), while knowledge access (β = .183, SE = .099, *p* = .064) and inhibitory control (β = -.194, SE = .101, *p* = .056) positively predicted development of children’s lying from T2 to T3. Surprisingly, children’s lying negatively predicted development of belief-emotion from T2 to T3 (β = -.255, SE = .098, *p* = .009)

# Supplementary Figures

**Figure S1**

*Schematic representation of the four competing models*

*Model 1: Stability model*

ToM/EF

Time 1

Time 2

Time 3

ToM/EF

ToM/EF

Lying

Lying

Lying

*Model 2: ToM/EF-to- lying model*

ToM/EF

Time 1

Time 2

Time 3

ToM/EF

ToM/EF

Lying

Lying

Lying

*Model 3: Lying-to- ToM/EF model*

ToM/EF

Time 1

Time 2

Time 3

ToM/EF

ToM/EF

Lying

Lying

Lying

*Model 4: Reciprocal model*

ToM/EF

Time 1

Time 2

Time 3

ToM/EF

ToM/EF

Lying

Lying

Lying

**Figure S2**

*Standardized estimates for significant paths in the reciprocal model after controlling for children’s age and verbal ability*

Diverse desire

Lying

Knowledge access

Lying

Knowledge access

Lying

T1

T3

T2

Belief-emotion

.230*(.092)

.363***(.091)

.183^#^(.099)

.237*(.098)

Inhibitory control

Inhibitory control

Inhibitory control

.293**(.100)

-.194^#^(.101)

-.255**(.098)

.195^#^(.104)

.285**(.101)

Belief-emotion

.274**(.095)

.228*(.092)

*Note.* Synchronous correlations are omitted from this figure, but were included in the analyses.

****p* < .001; ***p* < .01; **p* < .05; ^#^*p* <.08.
